# The Association between Dietary Quality and Dietary Guideline Adherence with Mental Health Outcomes in Adults: A Cross-Sectional Analysis

**DOI:** 10.3390/nu9030238

**Published:** 2017-03-05

**Authors:** Amy P. Meegan, Ivan J. Perry, Catherine M. Phillips

**Affiliations:** 1 HRB Centre for Diet and Health Research, School of Public Health, Physiotherapy and Sports Science, University College Dublin, Belfield, Dublin 4, Ireland; amy.meegan@ucdconnect.ie; 2 HRB Centre for Diet and Health Research, Department of Epidemiology and Public Health, University College Cork Western Gateway Building, Western Rd, Cork, Ireland; i.perry@ucc.ie

**Keywords:** mental health, depression, anxiety, well-being, dietary quality, Mitchelstown cohort, cross-sectional study

## Abstract

The prevalence of adverse mental health outcomes in adults is increasing. Although beneficial effects of selected micronutrients and foods on mental health have been reported, they do not reflect the impact of the habitual diet on mental health. Therefore, our objective is to examine potential associations between dietary quality, dietary composition and compliance with food pyramid recommendations with depressive symptoms, anxiety and well-being (assessed using CES-D, HADS-A and WHO-5 screening tools) in a cross-sectional sample of 2047 middle-aged adults. Diet was assessed using a self-completed FFQ. Chi-square tests, *t*-tests and logistic regression analyses were used to investigate the associations between dietary components and mental health outcomes. Dietary quality, but not dietary composition or guideline adherence, was associated with well-being. Those with high dietary quality were more likely to report well-being (OR =1.67, 95% CI 1.15–2.44, *p* = 0.007) relative to those with low dietary quality. This remained significant among females (OR = 1.92, (95% CI 1.14–3.23, *p* = 0.014) and non-obese individuals (OR = 2.03, 95% CI 1.28–3.20, *p* = 0.003). No associations between any dietary measures with anxiety or depressive symptoms were observed. These novel results highlight the importance of dietary quality in maintaining optimal psychological well-being. Better understanding of the relationship between dietary quality and mental health may provide insight into potential therapeutic or intervention strategies to improve mental health and well-being.

## 1. Introduction

More than a quarter of the adult European population have experienced a mental health disorder [1]. The development of psychological health is a multi-factorial process involving genetic, biological, social and environmental factors. In recent years, attention has turned to the potential role of modifiable lifestyle behaviours, such as diet, in the development of common mental health disorders. Studies examining diet-mental health relationships focused on specific micronutrients such as folate and B-vitamins [2,3,4], macronutrients such as fatty acids [5,6], and single food items and groups such as tea, fruit, vegetables and fish [6,7,8] have added to the knowledge base. However, observing the effect of individual nutrients and foods may not be representative of the impact of whole diet on mental health, as diet is a complex combination of foods and nutrients which are not consumed in isolation. Methods which examine the combined effects of multiple dietary components, and thereby reflect the real-life scenario, could have important public health implications because the messages on habitual whole diet intake, rather than specific nutrients may be more communicable to public health. Furthermore dietary pattern analysis may be a more realistic predictor of disease risk [9]. 

Inconsistent data regarding the relationship between dietary patterns and dietary quality with risk of adverse mental health outcomes exists [10,11,12,13,14]. Moreover two recent systematic reviews have provided limited and conflicting evidence supporting the association between dietary patterns and depression in adults [15,16]. Furthermore a separate review evaluating the impact of randomised controlled dietary interventions (with a whole-of-diet approach) on depression and anxiety revealed some evidence for dietary interventions improving depression, but not anxiety [17]. It is likely that differences between studies such as how dietary exposures and mental health outcomes are defined, together with study design aspects including sample size, gender balance, age range and determination of confounding factors may contribute to the reported disparity. The majority of studies examining the association between dietary markers and mental health outcomes have focussed on the presence of depressive symptoms and/or anxiety as a marker of poor mental health. Screening tools for anxiety and depressive symptoms in adults include the Beck Anxiety Inventory, the Beck Depression Inventory-II, the Patient Health Questionnaire-9, the Hospital Anxiety and Depression Scale (HADS) and the Centre for Epidemiologic Studies Depression Scale (CES-D) [18,19,20]. Limited research has been conducted on the association between dietary indices and psychological well-being using the World Health Organisation (WHO)-5 Well Being Index [21]. Derived from a short questionnaire measuring subjective well-being, the WHO-5 has adequate validity both as a screening tool for depressive symptoms and as an outcome measure in clinical trials [22]. Therefore the primary objective of the present study was to examine diet-mental associations by examining the relationship between dietary quality, dietary composition and compliance with food pyramid recommendations with depressive symptoms, anxiety and emotional well-being, assessed using the CES-D, HADS-A and WHO-5 tools, in a cross-sectional sample of middle-aged adults. Given the reported influence of gender and obesity on mental health [23,24], secondary objectives included examination of diet-mental health associations in subgroups defined by gender and BMI status.

## 2. Subjects and Methods

### 2.1. Study Design and Subject Recruitment

The Cork and Kerry Diabetes and Heart Disease Study (Phase II) was a single centre, cross-sectional study conducted between 2010 and 2011 [25]. A population representative random sample was recruited from a large primary care centre in Mitchelstown, County Cork, Ireland (Mitchelstown cohort). The Livinghealth Clinic includes eight general practitioners and serves a catchment area of approximately 20,000 with a mix of urban and rural residents. Mitchelstown cohort participants were randomly selected from all registered attending patients in the 50–69 year age group. In total 3807 potential participants were selected from the practice list. Following exclusion of duplicates, deaths and ineligibles, 3043 were invited to participate in the study and of these 2047 Caucasian individuals (49.2% male) completed the questionnaire and physical examination components of the baseline assessment (response rate 67%). Ethics committee approval conforming to the Declaration of Helsinki was obtained from the Clinical Research Ethics Committee of University College Cork (reference ECM 4 (aa) 02/02/10) All participants provided written informed consent. Following exclusion of individuals without body mass index (BMI) data the remaining 2040 participants were used in the analyses.

### 2.2. Clinical and Anthropometric Data 

All participants attended the clinic in the morning after an overnight fast (minimum 8 h). Fasting blood samples were taken on arrival. Participants completed a General Health Questionnaire (GHQ) and a Food Frequency Questionnaire (FFQ) and the International Physical Activity Questionnaire (IPAQ). Data on age, gender, family history, medication use and medical history was gathered through a self-completed GHQ. Depressive symptoms, anxiety and well-being were assessed using a range of questionnaires including the 20-item Centre for Epidemiologic Studies Depression Scale (CES-D) [18], designed to evaluate the frequency and severity of depressive symptoms, the Hospital Anxiety and Depression Scale (HADS), using only the anxiety subscale [20] and the World Health Organisation (WHO)-5 Well Being Index [21]. Subjects with CES-D scores ≥ 16, HADS scores ≥ 13 and WHO-5 scores of >13 and were identified as having depressive symptoms, anxiety and good well-being, respectively. The WHO-5 Well-being index includes five items which are rated on a 6-point Likert scale from 0 (=not present) to 5 (=constantly present). The specific statements are (1) I have felt cheerful and in good spirits; (2) I have felt calm and relaxed; (3) I have felt active and vigorous; (4) I woke up feeling fresh and rested; and (5) My daily life has been filled with things that interest me. Data regarding antidepressant medication use were collected. History of depression and anxiety was assessed using the following questions: “Have you ever had depression?” “Have you ever had anxiety?” Subjects were then asked “If yes, when did it start? In the last year/1–5 years ago/>5 years ago.” Anthropometric measurements were recorded with calibrated instruments according to a standardised protocol. Body weight was measured in kilograms without shoes; to the nearest 100 g using a Tanita WB100MA weighing scales (Tanita Corporation, IL, USA). Height was measured in centimetres to one decimal place using a Seca Leicester height gauge (Seca, Birmingham, UK). Waist circumference (defined as mid-way between lowest rib and iliac crest) was measured in centimetres to one decimal place using a Seca 200 measuring tape (Seca, Birmingham, UK). The average of two measures was used for analyses. BMI was calculated. Individuals with a BMI ≥ 30 kg/m^2^ were defined as obese.

### 2.3. Dietary and Lifestyle Data

Diet was assessed using a modified version of the self-completed EPIC FFQ [26], which was originally validated using food diaries and a protein biomarker in a volunteer sample [27]. This FFQ was then incorporated into the Irish National Surveys of Lifestyle Attitudes and Nutrition 1998, 2002, 2006 [28,29,30] and the Cork and Kerry Phase 1 study [31] and has been validated for use in the Irish population. Information on the frequency of consumption of food items during the past 12 months was collected. The daily intake of energy and nutrients was computed from FFQ data using a tailored computer program (FFQ_Software Ver. 1.0; developed by the National Nutrition Surveillance Centre, School of Public Health, Physiotherapy and Population Science, University College Dublin, Belfield, Dublin 4, Ireland), which linked frequency selections with the food equivalents in McCance and Widdowson Food Tables [32]. A modified USDA Food Pyramid (to include foods commonly eaten in Ireland) was used to determine daily number of servings from each food pyramid shelf [33]. Serving sizes for each food type were included on the FFQ. Compliance with food pyramid recommendations was defined as >median score based on meeting each shelf recommendation (≥6 servings of bread, cereal, rice or pasta, ≥5 servings of fruit and vegetables, =3 servings of dairy, =2 servings of meat, fish or poultry, ≤3 servings of oils, fats and confectionary).

A dietary score (the DASH score) was calculated using these FFQ responses. It was a composite score derived from standard food groups within the FFQ. For each food group, consumption was divided into quintiles and participants were classified according to their intake ranking [34].Consumption of healthy food components were rated on a scale of 1–5, the higher the score the more frequent the consumption of that food, i.e., those in quintile one had the lowest consumption and received a score of one; conversely those in quintile five had the highest consumption and received a score of five. Less healthy dietary constituents, where low consumption is desired, were scored on a reverse scale with lower consumption receiving the higher scores. Component scores were summed and an overall DASH score for each person was calculated (ranging from 13 to 45)—a lower score indicated a poorer dietary quality. Furthermore, the DASH score was categorised into high (>median) and low (<median) dietary quality. 

Physical activity levels were assessed using the short form IPAQ which provided information on frequency, duration and intensity of physical activity [35]. Using the instrument's scoring protocol, moderate physical activity was defined according to frequency of activity, duration of each activity bout and metabolic equivalent (MET) minutes per week. Questions regarding smoking status allowed us to define current smokers as those smoking at present. Alcohol consumption included questions based on weekly intake to define moderate drinkers (women and men consuming less than 14 units and 21 units, respectively, in a typical week).

### 2.4. Statistical Analysis

Statistical analysis was conducted using PASW Statistics version 20 for Windows (SPSS Inc., Chicago, IL, USA). Continuous variables were expressed as means ± standard error of the mean (SEM) and categorical variables as percentages. Variables were assessed for normality of distribution and skewed variables were normalised as appropriate. Differences between groups were analysed by independent samples *t*-tests for continuous variables and by Chi-Square test for categorical variables. Logistic regression determined the relationship between dietary quality and food pyramid compliance with depressive symptoms, anxiety and well-being in the cohort and subgroups stratified by gender and by BMI. Antidepressant use, history of depression, age, gender, BMI, physical activity, smoking status and alcohol consumption were considered confounding factors. For all analyses a *p*-value of <0.05 was considered significant.

## 3. Results

### 3.1. Demographic, Dietary and Lifetsyle Characteristics According to Mental Health Status

Demographic, dietary and lifestyle characteristics of the entire cohort (*n =* 2040) and stratified by presence of depressive symptoms and anxiety, and well-being status, are presented in Table 1. Prevalence of depressive symptoms and anxiety were 16.1% and 15% respectively and 19.9% of the cohort did not report well-being. Individuals displaying depressive symptoms and not reporting well-being were younger than their respective counterparts. In addition those with depressive symptoms had greater BMI and anthropometric measures. There were no significant demographic or clinical differences when stratified by anxiety. 

Regarding dietary and lifestyle factors, we examined dietary intake and composition, food pyramid servings, compliance to food pyramid recommendations and dietary quality, moderate physical activity, alcohol consumption and smoking status according to mental health status. Although few differences with respect to dietary composition were observed, dietary quality was significantly higher among individuals reporting well-being compared to their respective counterparts. Examination of daily servings from each shelf of the food pyramid revealed lower consumption of breads, cereals, rice and pasta, meat, fish and poultry as well as less oils, fats and confectionary among those reporting anxiety. Daily fruit and vegetable consumption was higher among those reporting well-being. Overall compliance to food pyramid recommendations was not different between any groups. Scores for depressive symptoms, anxiety and well-being were analysed according to compliance with food pyramid recommendations and dietary quality. No differences in either the CESD or HADS-A scores were noted when stratified by these dietary measures (data not shown). However the WHO-5 well-being score was significantly higher among individuals with high dietary quality in comparison those with low dietary quality (17.2 ± 0.20 vs. 16.6 ± 0.20, *p* = 0.04 respectively). 

Regarding lifestyle behaviours, less engagement in moderate physical activity was observed among those displaying depressive symptoms and those who did not report well-being. Smoking and alcohol behavior did not differ according to mental health status.

### 3.2. Dietary Quality, Food Pyramid Compliance and Mental Health Outcomes

Logistic regression analysis further examined the association between mental health outcomes and dietary quality and compliance to food pyramid recommendations (Table 2). Results of the fully adjusted logistic regression model revealed that the likelihood of well-being was greater among those with high dietary quality OR = 1.67, 95% CI 1.15–2.43, *p* = 0.007) among the entire cohort. Neither the risk of depressive symptoms (OR = 1.06, 95% CI 0.69–1.63, *p* = 0.800) nor anxiety (OR = 0.77, 95% CI 0.52–1.16, *p* = 0.217) was significantly reduced. Compliance with food pyramid recommendations was not significantly associated with any of the mental health outcomes. 

### 3.3. Influence of Gender and BMI on Association between Dietary Quality and Mental Health Status

Examination of whether gender modulated the observed association between dietary quality and mental health parameters revealed some gender specific findings (Table 3). Females with high dietary quality were almost twice as likely to report well-being (OR = 1.92, 95% CI 1.14–3.23, *p* = 0.014) compared to those with low dietary quality. No association between dietary quality and the other mental health outcomes were observed, nor were any diet-mental health associations noted among the male participants. Stratification of the cohort according to BMI status (Table 4) revealed that the association between high dietary quality and well-being remained significant in the non-obese subjects only, even in the fully adjusted model. Among those with high dietary quality the likelihood of well-being was two-fold greater among the non-obese individuals (OR = 2.04, 95% CI 1.28–3.23, *p* = 0.003), relative to their non-obese counterparts with low dietary quality. There was a non-significant but positive association between high dietary quality and reduced risk of anxiety among obese subjects after final adjustments were made for antidepressant use and history of depression (OR = 0.44, 95% CI 0.19–1.02, *p =* 0.056). No significant associations were evident between dietary quality and depressive symptoms in the stratified analysis. 

## 4. Discussion 

As the prevalence of psychological disorders continues to grow, evidence of modifiable lifestyle behaviours and interventions which favourably impact mental health is essential. To our knowledge, the current study is the first to examine dietary composition, dietary quality and compliance with food pyramid recommendations and their association with depressive symptoms, anxiety and well-being in an adult population. We provide evidence for an association between high dietary quality and good psychological well-being. Those with high dietary quality were almost twice as likely to report good well-being, even after adjustments for confounding factors, including antidepressant use and history of depression. When stratified by gender and BMI, the association between dietary quality and well-being was evident among both the female and non-obese study participants.

The reasons why high dietary quality was associated with better well-being, but not reduced risk of depressive symptoms or anxiety, remain uncertain. Healthy dietary patterns such as the Mediterranean and Norwegian diets, which are characterised by a high intake of fruit, vegetables, wholegrains, fish and meat, have been associated with reduced risk of depressive symptoms [15,16]. The Western or unhealthy diet, characterised by higher intakes of refined grains, processed meat, high-fat products, high-sugar products and alcohol, has been associated with increased risk of depression [36,37]. However systematic reviews and meta-analysis have not provided confirmation, but this may have been due to too few studies [15,16]. Dietary patterns and quality have also been linked with risk of anxiety and stress [38,39,40], suggesting that the mechanisms underlying diet-mental health associations are not limited to depressive symptoms. Data from genome-wide association studies have provided insights into the genetic architecture of psychiatric disorders and suggest the presence of many genetic loci each with a small effect influencing susceptibility to depression and anxiety [41]. It is therefore reasonable to hypothesise that the lack of association between dietary quality and risk of depressive symptoms and anxiety in the present study may be due to non-modifiable genetic influences which predispose individuals to depressive symptoms and anxiety. Well-being is a non-heritable trait, thus it may be possible to improve an individual’s state of well-being by altering lifestyle, environmental and/ or dietary factors, such as dietary quality. It is noteworthy that compliance with food pyramid recommendations was not associated with well-being. It may be that individuals with high dietary quality consumed healthy foods but not in the proportions recommended by national dietary guidelines. For example, an individual may consume only one portion of dairy per day (non-compliant with food pyramid recommendations) but the remainder of their diet may score highly on the DASH scoring system. 

Mental health disorders, such as depression and anxiety, are more common among women than men. Gender differences have been reported in age of onset of symptoms, frequency and duration of symptoms, social adjustment and long term outcome [23]. The present study demonstrates an association between high dietary quality and improved well-being, which was only evident in the stratified analysis among the female participants. Consistent with these findings, data from the Wellbeing, Eating and Exercise for a Long Life (WELL) study also indicated gender differences with regard to the association between dietary quality (assessed by the dietary guideline index and recommended food score) and well-being [42]. Similarly, results from the Hordaland Study revealed that high dietary quality was associated with reduced risk of both depression and anxiety in women but only reduced risk of depression in men [38]. Although speculative, one plausible explanation may be that the greater sense of well-being observed among the women in the current study may have been, at least partly, driven by their weight and appearance. Both BMI and waist circumference were lower among the women with high dietary quality compared to their counterparts with low dietary quality (*p* = 0.09 and *p* = 0.02, respectively). Although the association between dietary quality and well-being was non-significant among males, the direction of the association was the same as that observed among females. The notion that men are less concerned about their weight and health may provide some explanation for these findings. It is noteworthy that among those with reduced well-being, 45% were men and 55% were women. Considering the unequal gender balance it is possible that the females are drivers of the association between higher dietary quality and improved well-being which was observed when the entire cohort was examined.

The increasing prevalence of both adverse mental health and obesity over recent decades, which account for a large proportion of the global burden of disease, represents a major public health concern [43]. Data from the Australian PATH Through Life Project, a large community survey involving adults (aged 20–24 (*n* = 2280), 40–44 (*n* = 2334) and 60–64 years *n* = 2305)), demonstrated associations between obesity and depression, anxiety and emotional well-being in women, but not in men [44]. Furthermore greater odds ratios for poor well-being (up to 1.72, 95% CI 1.26–2.36) among obese individuals, especially those with BMI > 40kg/m^2^ have been reported in the Health Survey for England [24]. Interestingly, being underweight was negatively associated with well-being, whereas being overweight but not obese was positively associated with well-being. These data suggest a U-shaped association between BMI and well-being, implying a protective effect of a BMI ranging from 18 to 25 kg/m^2^ on emotional well-being. Data regarding obesity-mental health associations which take diet into account are scarce. In the current work we performed stratified analysis examining the relationship between dietary measures and mental health according to BMI status. The association between dietary quality and well-being observed in the entire cohort remained significant in the non-obese subjects only, even in the fully adjusted models. No associations between any dietary measure and either depressive symptoms or anxiety were noted among the obese individuals. Although it has been suggested that the inter-relationship between obesity and adverse mental health is bidirectional [43,45], the evidence base is inconsistent [46,47,48,49]. Moreover Jacka et al., recently examined whether reverse causality can explain the relationship between diet and mental health [50]. Thus it is clear that the inter-relationships between diet, obesity and mental health are highly complex. Future prospective cohort studies examining these relationships, with a focus on the reverse causality hypothesis, are required to further establish direction of association and underlying mechanisms.

The present study had several strengths including a large sample size of participants aged 50 to 69 years old, of which 49.2% were male. The inclusion of questionnaires to assess mental health parameters, diet and lifestyle factors were important for providing a comprehensive assessment of depressive symptoms, anxiety and well-being and a wide range of confounding factors, including history of depression and use of antidepressants. Furthermore, the on-going follow-up of the Mitchelstown cohort will allow longitudinal analysis of mental health outcomes and associations with diet, gender and BMI in an aging population. Notwithstanding these strengths, the current work must be considered within context with its limitations. The cross-sectional study design limited our ability to draw a conclusion about the causal relationship between dietary quality and mental health with BMI. Mental illness can affect appetite and influence food choices, for example increased consumption of fast food [51] and energy dense sweets [52] in conjunction with reduced intake of fruits and vegetables [53]. Thus prospective studies to determine if poor dietary quality is a result of reduced well-being rather than a causative factor are warranted. It is therefore not possible to exclude reverse causality as an explanation for the present results. Additionally the presence of residual confounding by socio-economic and/or behavioural factors, or unmeasured confounding by factors such as personality traits may also, at least in part, account for some of our findings. Furthermore as suggested by Kraemer et al., caution should be exercised in interpreting OR as effect size in cohort studies where the outcomes are common (>10% of population), as the more frequent the outcome the more the odds ratio may over/under estimate the risk ratio [54]. Finally, the generalisability of our findings may be limited. The Mitchelstown cohort was a random sample of middle-aged adults recruited from a large primary care centre (Livinghealth Clinic) in Mitchelstown, County Cork, Ireland, which includes eight general practitioners and serves a catchment area of approximately 20,000. Previous research suggests that approximately 98% of Irish adults are registered with a general practitioner and that, even in the absence of a universal patient registration system, it is possible to perform population based epidemiological studies that are representative of the general population using these methods [55].

## 5. Conclusions

Poor mental health has a significant economic and societal impact [56]. Based on the growing prevalence and negative health outcomes associated with poor mental health, there is an undeniable need for inexpensive and effective strategies to reduce the risk and burden of mental health problems, ultimately leading to an improvement in the psychological well-being of the population. Our data support the hypothesis that high dietary quality is associated with good emotional well-being. This association was particularly evident among females and non-obese middle-aged adults, following adjustments for confounding factors. These findings are of public health and clinical relevance as they highlight the potential of dietary interventions for improving psychological well-being and the importance of risk stratification, in terms of distinguishing population groups that may be at greater risk of adverse mental health. From a public health perspective, improving dietary quality appears to be a logical and feasible avenue to pursue in the primary prevention of adverse mental health. Confirmation of a causal relationship between dietary quality and mental health may have potential downstream benefits in risk stratification for mental health disorders and in formulating appropriate targeted therapeutic and intervention approaches, especially for those who have failed to respond to pharmacological and psychological treatment. Thus future prospective cohort studies and controlled trials examining the potential of increasing dietary quality for improving mental health and well-being are warranted. Indeed recent data from the SMILES trial (a randomized controlled trial of dietary improvement for adults with major depression) are very encouraging as they provide the first preliminary evidence that personalised one-to-one dietary counseling may be a suitable option to consider in the delivery and management of individuals with adverse mental health disorders [57]. Future nutritional psychiatry research should aim to replicate and confirm these findings.

## Figures and Tables

**Table 1:** Demographic, dietary and lifestyle characteristics of the Mitchelstown cohort according to depressive symptoms, anxiety and well-being status.

Characteristics	Entire cohort	Depressive Symptoms			Anxiety			Well-being		
		Yes	No	*p*	Yes	No	*p*	No	Yes	*p*
*n*	2040	302	1572		276	1561		383	1541	
Age (years)	59.8 ± 0.1	58.9 ± 0.3	59.7 ± 0.1	0.046	59.8 ± 0.3	59.8 ± 0.1	0.931	58.2 ± 0.3	60.0 ± 0.1	0.000
Gender (% male)	49.2	44.0	50.7	0.034	51.1	51.3	0.462	45.4	49.6	0.146
BMI (kg/m^2^)	28.6 ± 0.1	29.4 ± 0.3	28.4 ± 0.1	0.002	28.6 ± 0.3	28.6 ± 0.1	0.990	28.9 ± 0.3	28.5 ± 0.1	0.112
Waist (cm)	97,0 ± 0.3	98.3 ± 0.8	96.6 ± 0.3	0.034	97.5 ± 0.7	97.1 ± 0.3	0.641	97.4 ± 0.7	96.8 ± 0.3	0.442
Hip (cm)	100.4 ± 0.2	102.1 ± 0.6	99.9 ± 0.3	0.001	100.7 ± 0.6	100.5 ± 0.3	0.702	100.9 ± 0.5	100.3 ± 0.3	0.295
***Dietary composition & dietary quality***										
Kilocalories (kcal)	2034 ± 18	2044 ± 48	2022 ± 20	0.668	1960 ± 49	2052 ± 21	0.091	2034 ± 41	2034 ± 21	0.989
Fat (% EI)	33.74 ± 0.15	33.73 ± 0.39	33.82 ± 0.18	0.838	34.10 ± 0.40	33.72 ± 0.18	0.398	34.08 ± 0.36	33.71 ± 0.18	0.366
Carbohydrates (% EI)	48.97 ± 0.19	49.19 ± 0.47	48.77 ± 0.22	0.429	48.04 ± 0.49	49.13 ± 0.22	0.049	48.96 ± 0.42	48.88 ± 0.22	0.882
Protein (% EI)	18.6 ± 0.09	18.70 ± 0.24	18.58 ± 0.11	0.633	19.05 ± 0.29	18.50 ± 0.11	0.048	18.65 ± 0.20	18.58 ± 0.11	0.765
Fibre (% EI)	2.60 ± 0.03	2.67 ± 0.04	2.57 ± 0.02	0.050	2.58 ± 0.05	2.61 ± 0.02	0.646	2.61 ± 0.04	2.58 ± 0.02	0.511
DASH score	28.85 ± 0.15	28.92 ± 0.38	28.89 ± 0.17	0.930	28.59 ± 0.41	28.89 ± 0.17	0.506	28.25 ± 0.34	29.04 ± 0.17	0.037
***Daily servings from food pyramid shelves & compliance to recommendations***										
Bread, cereal, rice & pasta	5.27 ± 0.07	5.28 ± 0.17	5.32 ± 0.08	0.843	4.73 ± 0.14	5.28 ± 0.08	0.004	5.20 ± 0.16	5.32 ± 0.08	0.490
Fruit & vegetable	7.14 ± 0.11	6.78 ± 0.26	7.18 ± 0.13	0.212	7.08 ± 0.29	7.17 ± 0.14	0.789	6.61 ± 0.20	7.34 ± 0.14	0.014
Dairy	1.94 ± 0.03	1.97 ± 0.09	1.96 ± 0.04	0.851	1.80 ± 0.09	1.94 ± 0.04	0.143	2.01 ± 0.08	1.94 ± 0.04	0.408
Meat, fish or poultry	2.40 ± 0.03	2.50 ± 0.08	2.38 ± 0.03	0.163	2.24 ± 0.06	2.42 ± 0.03	0.033	2.42 ± 0.06	2.39 ± 0.03	0.644
Oils, fats & confectionary	7.89 ± 0.11	7.98 ± 0.30	7.96 ± 0.13	0.963	7.29 ± 0.26	7.97 ± 0.13	0.041	7.93 ± 0.25	7.90 ± 0.13	0.918
High compliance (%) ^1^	46.4	49.0	45.9	0.326	46.7	45.3	0.656	46.2	46.2	0.997
***Lifestyle Factors***										
Physically active (%) ^2^	29.5	21.9	31.7	0.000	29.5	29.6	0.930	25.2	30.6	0.000
Alcohol consumers (%) ^3^	65.2	66.2	64.7	0.702	67.2	64.6	0.596	69.0	63.8	0.264
Current smokers (%)	14.7	17.1	14.3	0.434	16.3	14.6	0.775	16.5	14.2	0.448

Continuous variables are expressed as means ± SEM and categorical variables as percentages. Independent samples *t*-tests are used for continuous variables and Chi-Square test are used for categorical variables. Yrs: years; %: percentage; kg: kilograms; m^2^: metre squared; cm: centimetre; EI; energy intake. ^1^ High compliance defined as >median score for compliance to food pyramid recommendations. ^2^ Physically active defined as having a moderate level of physical activity. ^3^ Moderate alcohol consumers.

**Table 2:** Odds ratios (95% CI) for depressive symptoms, anxiety and well-being according to dietary quality and compliance with food pyramid recommendations among the Mitchelstown cohort.

	Depressive Symptoms		Anxiety		Well-Being	
Model 1		*p*		*p*		*p*
Low dietary quality/compliance	1 [reference]		1 [reference]		1 [reference]	
High dietary quality	0.84 (0.63–1.13)	0.25	0.83 (0.61–1.14)	0.25	1.61 (1.22–2.13)	0.001
High compliance	1.12 (0.88–1.44)	0.36	1.01 (0.83–1.38)	0.62	0.99 (0.79–1.25)	0.94
Model 2						
Low dietary quality/compliance	1 [reference]		1 [reference]		1 [reference]	
High dietary quality	1.06 (0.69–1.63)	0.80	0.77 (0.52–1.16)	0.22	1.67 (1.15–2.44)	0.007
High compliance	1.32 (0.92–1.90)	0.13	1.23 (0.87–1.73)	0.24	1.11 (0.81–1.52)	0.53

Data is presented as OR (95% CI). DASH score and compliance to food pyramid recommendation score were stratified by median to define low/high dietary quality and compliance, respectively. Reference group refers to that within the same comparative group. Model 1: Adjusted for age, gender and BMI. Model 2: Additionally adjusted for smoking, physical activity, alcohol consumption, antidepressant use and history of depression.

**Table 3:** Odds ratios (95% CI) for mental health outcomes according to dietary quality stratified by gender.

	Depressive Symptoms		Anxiety		Well-Being	
Model 1		*p*		*p*		*p*
Low Dietary Quality	1 [reference]		1 [reference]		1 [reference]	
High Dietary Quality (Male)	0.87 (0.55–1.38)	0.56	0.99 (0.64–1.52)	0.95	1.35 (0.89–2.08)	0.16
High Dietary Quality (Female)	0.82 (0.56–1.20)	0.30	0.71 (0.46–1.10)	0.12	1.85 (0.90–2.63)	0.001
Model 2						
Low Dietary Quality	1 [reference]		1 [reference]		1 [reference]	
High Dietary Quality (Male)	0.90 (0.46–1.70)	0.77	0.91 (0.53–1.58)	0.75	1.37 (0.79–2.38)	0.27
High Dietary Quality (Female)	1.25 (0.69–2.29)	0.46	0.62 (0.34–1.14)	0.13	1.92 (1.14–3.23)	0.014

Data is presented as OR (95% CI). DASH score was stratified by median to define low/high dietary quality. Reference group refers to that within the same comparative group. Model 1: Adjusted for age and BMI. Model 2: Additionally adjusted for smoking, physical activity, alcohol consumption, antidepressant use and history of depression.

**Table 4:** Odds ratios (95% CI) for mental health outcomes according to dietary quality stratified by BMI status.

	Depressive Symptoms		Anxiety		Well-Being	
Model 1		*p*		*p*		*p*
Low dietary quality	1 [reference]		1 [reference]		1 [reference]	
High dietary quality (Obese)	1.13 (0.70–1.82)	0.63	0.64 (0.37–1.12)	0.12	1.27 (0.79–2.00	0.34
High dietary quality (Non-obese)	0.72 (0.49–1.05)	0.08	0.94 (0.65–1.37)	0.75	1.79 (1.28–2.56)	0.001
Model 2						
Low dietary quality	1 [reference]		1 [reference]		1 [reference]	
High dietary quality (Obese)	1.57 (0.74–3.30)	0.237	0.44 (0.19–1.02)	0.056	1.16 (0.58–2.33)	0.671
High dietary quality (Non-obese)	0.83 (0.48–1.43)	0.497	0.92 (0.57–1.48)	0.728	2.04 (1.28–3.23)	0.003

Data is presented as OR (95% CI). DASH score was stratified by median to define low/high dietary quality. Reference group refers to that within the same comparative group. Model 1: Adjusted for age and gender. Model 2: Additionally adjusted for smoking, physical activity, alcohol consumption, antidepressant use and history of depression.

## Abbreviations

CES-D	Centre for Epidemiologic Studies Depression Scale
HADS	Hospital Anxiety and Depression Scale
WHO-5	World Health Organization-5 Well Being Index
FFQ	Food Frequency Questionnaire
OR	Odds Ratio.

## References

[1] World Health Organisation Mental Health: Data and Statistics. http://www.euro.who.int/en/health-topics/noncommunicable-diseases/mental-health/data-and-statistics.

[2] Sanchez-Villegas A., Doreste J., Schlatter J., Pla J., Bes-Rastrollo M., Martinez-Gonzalez M.A. (2009). Association between folate, vitamin B(6) and vitamin B(12) intake and depression in the sun cohort study. J. Hum. Nutr. Diet..

[3] Tolmunen T., Hintikka J., Ruusunen A., Voutilainen S., Tanskanen A., Valkonen V.P., Viinamaki H., Kaplan G.A., Salonen J.T. (2004). Dietary folate and the risk of depression in finnish middle-aged men. A prospective follow-up study. Psychother. Psychosom..

[4] Tolmunen T., Hintikka J., Voutilainen S., Ruusunen A., Alfthan G., Nyyssonen K., Viinamaki H., Kaplan G.A., Salonen J.T. (2004). Association between depressive symptoms and serum concentrations of homocysteine in men: A population study. Am. J. Clin. Nutr..

[5] Daley C., Patterson A., Sibbritt D., MacDonald-Wicks L. (2015). Unsaturated fat intakes and mental health outcomes in young women from the australian longitudinal study on women’s heath. Public Health Nutr..

[6] Jacka F.N., Pasco J.A., Williams L.J., Meyer B.J., Digger R., Berk M. (2013). Dietary intake of fish and pufa, and clinical depressive and anxiety disorders in women. Br. J. Nutr..

[7] Hintikka J., Tolmunen T., Honkalampi K., Haatainen K., Koivumaa-Honkanen H., Tanskanen A., Viinamaki H. (2005). Daily tea drinking is associated with a low level of depressive symptoms in the Finnish general population. Eur. J. Epidemiol..

[8] Rooney C., McKinley M.C., Woodside J.V. (2013). The potential role of fruit and vegetables in aspects of psychological well-being: A review of the literature and future directions. Proc. Nutr. Soc..

[9] Hu F.B. (2002). Dietary pattern analysis: A new direction in nutritional epidemiology. Curr. Opin. Lipidol..

[10] Sanchez-Villegas A., Delgado-Rodriguez M., Alonso A., Schlatter J., Lahortiga F., Serra Majem L., Martinez-Gonzalez M.A. (2009). Association of the mediterranean dietary pattern with the incidence of depression: The seguimiento universidad de navarra/university of navarra follow-up (sun) cohort. Arch. Gen. Psychiatry.

[11] Rahe C., Baune B.T., Unrath M., Arolt V., Wellmann J., Wersching H., Berger K. (2015). Associations between depression subtypes, depression severity and diet quality: Cross-sectional findings from the bidirect study. BMC Psychiatry.

[12] Lai J.S., Oldmeadow C., Hure A.J., McEvoy M., Byles J., Attia J. (2016). Longitudinal diet quality is not associated with depressive symptoms in a cohort of middle-aged Australian women. Br. J. Nutr..

[13] Lai J.S., Hure A.J., Oldmeadow C., McEvoy M., Byles J., Attia J. (2015). Prospective study on the association between diet quality and depression in mid-aged women over 9 years. Eur. J. Nutr..

[14] Akbaraly T.N., Sabia S., Shipley M.J., Batty G.D., Kivimaki M. (2013). Adherence to healthy dietary guidelines and future depressive symptoms: Evidence for sex differentials in the Whitehall II study. Am. J. Clin. Nutr..

[15] Quirk S.E., Williams L.J., O’Neil A., Pasco J.A., Jacka F.N., Housden S., Berk M., Brennan S.L. (2013). The association between diet quality, dietary patterns and depression in adults: A systematic review. BMC Psychiatry.

[16] Lai J.S., Hiles S., Bisquera A., Hure A.J., McEvoy M., Attia J. (2014). A systematic review and meta-analysis of dietary patterns and depression in community-dwelling adults. Am. J. Clin. Nutr..

[17] Opie R.S., O'Neil A., Itsiopoulos C., Jacka F.N. (2015). The impact of whole-of-diet interventions on depression and anxiety: A systematic review of randomised controlled trials. Public Health Nutr..

[18] Radloff L.S. (1977). The CES-D scale: Self-report depression scale for research in the general population. Appl. Psychcol. Meas..

[19] Smarr K.L., Keefer A.L. (2011). Measures of depression and depressive symptoms: Beck depression inventory-II (BDI-II), center for epidemiologic studies depression scale (CES-D), geriatric depression scale (GDS), hospital anxiety and depression scale (HADS), and patient health questionnaire-9 (PHQ-9). Arthritis Care Res..

[20] Zigmond A.S., Snaith R.P. (1983). The hospital anxiety and depression scale. Acta Psychiatr. Scand..

[21] WHO (1998). Wellbeing Measures in Health Care: The Depcare Project: Report on a Who Meeting Stockholm, København, Sweden, 12–13 February 1998.

[22] Topp C.W., Ostergaard S.D., Sondergaard S., Bech P. (2015). The who-5 well-being index: A systematic review of the literature. Psychother. Psychosom..

[23] Piccinelli M., Gomez Homen F. (1997). Gender Differences in the Epidemiology of Affective Disorders and Scizhophrenia.

[24] Stranges S., Samaraweera P.C., Taggart F., Kandala N.B., Stewart-Brown S. (2014). Major health-related behaviours and mental well-being in the general population: The health survey for england. BMJ Open.

[25] Kearney P.M., Harrington J.M., Mc Carthy V.J., Fitzgerald A.P., Perry I.J. (2013). Cohort profile: The cork and kerry diabetes and heart disease study. Int. J. Epidemiol..

[26] Riboli E., Elmstahl S., Saracci R., Gullberg B., Lindgarde F. (1997). The malmo food study: Validity of two dietary assessment methods for measuring nutrient intake. Int. J. Epidemiol..

[27] Harrington J. (1997). Validation of a Food Frequency Questionnaire as a Tool for Assessing Nutrient Intake. Master’s Thesis.

[28] Friel S., Nic Gabhainn S., Kelleher C.C. (1999). The National Health and Lifestyle Surveys: Survey of Lifestyle, Attitudes and Nutrition (SLAN) and the Irish Health Behaviour in School-Aged Children Survey (HBSC).

[29] Kelleher C.C., Nic Gabhainn S., Friel S., Corrigan H., Nolan G., Sixsmith J. (2003). The National Health and Lifestyle Surveys: Survey of Lifestyle, Attitudes and Nutrition (SLAN) and the Irish Health Behaviours in School-Aged Children Survey (HBSC).

[30] Morgan K., McGee H., Watson D., Perry I., Barry M., Shelley E. (2008). Slan 2007: Survey of Lifestyle, Attitudes & Nutrition in Ireland.

[31] Villegas R., Salim A., Collins M.M., Flynn A., Perry I.J. (2004). Dietary patterns in middle-aged irish men and women defined by cluster analysis. Public Health Nutr..

[32] Food Standards Agency (2002). MC Cance and Widdowson’s the Composition of Foods.

[33] Department of Health and Children (2006). The Food Pyramid.

[34] Fung T.T., Chiuve S.E., McCullough M.L., Rexrode K.M., Logroscino G., Hu F.B. (2008). Adherence to a dash-style diet and risk of coronary heart disease and stroke in women. Arch. Intern. Med..

[35] International Physical Activity Questionnaire Guidelines for Data Processing and Analysis of the International Physical Activity Questionnaire (IPAQ)-Short and Long Forms. Guidelines for Data Processing and Analysis of the International Physical Activity Questionnaire (IPAQ)-Short and Long Forms. http://www.ipaq.ki.se/scoring.pdf.

[36] Akbaraly T.N., Brunner E.J., Ferrie J.E., Marmot M.G., Kivimaki M., Singh-Manoux A. (2009). Dietary pattern and depressive symptoms in middle age. Br. J. Psychiatry.

[37] Sanchez-Villegas A., Toledo E., de Irala J., Ruiz-Canela M., Pla-Vidal J., Martinez-Gonzalez M.A. (2012). Fast-food and commercial baked goods consumption and the risk of depression. Public Health Nutr..

[38] Jacka F.N., Mykletun A., Berk M., Bjelland I., Tell G.S. (2011). The association between habitual diet quality and the common mental disorders in community-dwelling adults: The hordaland health study. Psychosom. Med..

[39] Jacka F.N., Pasco J.A., Mykletun A., Williams L.J., Hodge A.M., O’Reilly S.L., Nicholson G.C., Kotowicz M.A., Berk M. (2010). Association of western and traditional diets with depression and anxiety in women. Am. J. Psychiatry.

[40] Munoz M.A., Fito M., Marrugat J., Covas M.I., Schroder H., REGICOR and HERMES investigators (2009). Adherence to the mediterranean diet is associated with better mental and physical health. Br. J. Nutr..

[41] Demirkan A., Penninx B.W., Hek K., Wray N.R., Amin N., Aulchenko Y.S., van Dyck R., de Geus E.J., Hofman A., Uitterlinden A.G. (2011). Genetic risk profiles for depression and anxiety in adult and elderly cohorts. Mol. Psychiatry.

[42] Milte C.M., Thorpe M.G., Crawford D., Ball K., McNaughton S.A. (2015). Associations of diet quality with health-related quality of life in older Australian men and women. Exp. Gerontol..

[43] Gatineau M., Dent M. (2011). Obesity and Mental Health.

[44] Jorm A.F., Korten A.E., Christensen H., Jacomb P.A., Rodgers B., Parslow R.A. (2003). Association of obesity with anxiety, depression and emotional well-being: A community survey. Aust. N. Z. J. Public Health.

[45] Pan A., Sun Q., Czernichow S., Kivimaki M., Okereke O.I., Lucas M., Manson J.E., Ascherio A., Hu F.B. (2012). Bidirectional association between depression and obesity in middle-aged and older women. Int. J. Obes..

[46] Atlantis E., Baker M. (2008). Obesity effects on depression: Systematic review of epidemiological studies. Int. J. Obes..

[47] Fezeu L.K., Batty G.D., Gale C.R., Kivimaki M., Hercberg S., Czernichow S. (2015). Is the relationship between common mental disorder and adiposity bidirectional? Prospective analyses of a UK general population-based study. PLoS ONE.

[48] Kivimaki M., Batty G.D., Singh-Manoux A., Nabi H., Sabia S., Tabak A.G., Akbaraly T.N., Vahtera J., Marmot M.G., Jokela M. (2009). Association between common mental disorder and obesity over the adult life course. Br. J. Psychiatry.

[49] Kivimaki M., Lawlor D.A., Singh-Manoux A., Batty G.D., Ferrie J.E., Shipley M.J., Nabi H., Sabia S., Marmot M.G., Jokela M. (2009). Common mental disorder and obesity: Insight from four repeat measures over 19 years: Prospective Whitehall II cohort study. BMJ.

[50] Jacka F.N., Cherbuin N., Anstey K.J., Butterworth P. (2015). Does reverse causality explain the relationship between diet and depression?. J. Affect. Disord..

[51] Crawford G.B., Khedkar A., Flaws J.A., Sorkin J.D., Gallicchio L. (2011). Depressive symptoms and self-reported fast-food intake in midlife women. Prev. Med..

[52] Jeffery R.W., Linde J.A., Simon G.E., Ludman E.J., Rohde P., Ichikawa L.E., Finch E.A. (2009). Reported food choices in older women in relation to body mass index and depressive symptoms. Appetite.

[53] Konttinen H., Mannisto S., Sarlio-Lahteenkorva S., Silventoinen K., Haukkala A. (2010). Emotional eating, depressive symptoms and self-reported food consumption. A population-based study. Appetite.

[54] Kraemer H.C. (2006). Correlation coefficients in medical research: From product moment correlation to the odds ratio. Stat. Methods Med. Res..

[55] Hinchion R., Sheehan J., Perry I. (2002). Primary care research: Patient registration. Ir. Med. J..

[56] Gustavsson A., Svensson M., Jacobi F., Allgulander C., Alonso J., Beghi E., Dodel R., Ekman M., Faravelli C., Fratiglioni L. (2011). Cost of disorders of the brain in Europe 2010. Eur. Neuropsychopharmacol..

[57] Jacka F.N., O'Neil A., Opie R., Itsiopoulos C., Cotton S., Mohebbi M., Castle D., Dash S., Mihalopoulos C., Chatterton M.L. (2017). A randomised controlled trial of dietary improvement for adults with major depression (the ‘smiles’ trial). BMC Med..

